# Myocardial Infarction Susceptibility and the *MTNR1B* Polymorphisms

**DOI:** 10.3390/ijms241411444

**Published:** 2023-07-14

**Authors:** Ivana Škrlec, Zrinka Biloglav, Jasminka Talapko, Snježana Džijan, Danijela Daus-Šebeđak, Vera Cesar

**Affiliations:** 1 Faculty of Dental Medicine and Health, Josip Juraj Strossmayer University of Osijek, 31000 Osijek, Croatia; 2 Department of Medical Statistics, Epidemiology and Medical Informatics, School of Public Health Andrija Štampar, 10000 Zagreb, Croatia; 3 School of Medicine, University of Zagreb, 10000 Zagreb, Croatia; 4 DNA Laboratory, Genos Ltd., 10000 Zagreb, Croatia; 5 Family Medicine Practice ‘Dr. Danijela Daus-Šebeđak’, 35107 Podvinje, Croatia; 6 Department of Biology, Josip Juraj Strossmayer University of Osijek, 31000 Osijek, Croatia

**Keywords:** cardiovascular diseases, melatonin receptor, myocardial infarction, *MTNR1B*

## Abstract

Melatonin is a circadian hormone with antioxidant properties that protects against myocardial ischemia-reperfusion injury. Genetic variations of the melatonin receptor 1B gene (*MTNR1B*) play an important role in the development of type 2 diabetes, a risk factor for cardiovascular diseases. Accordingly, *MTNR1B* polymorphisms are crucial in numerous disorders of the cardiovascular system. Therefore, the aim of the present study was to investigate a possible association of *MTNR1B* polymorphisms with chronotype and susceptibility to myocardial infarction. The present case-control study included 199 patients with myocardial infarction (MI) (57% men) and 198 control participants (52% men) without previous cardiovascular diseases who underwent genotyping for the *MTNR1B* polymorphisms rs10830963, rs1387153, and rs4753426 from peripheral blood samples. Chronotype was determined using the Morningness-Eveningness Questionnaire (MEQ). As estimated by the chi-square test, no significant association was found in the distribution of alleles and genotypes between myocardial infarction patients and controls. In addition, there was no association between *MTNR1B* polymorphisms and chronotype in MI patients. As some previous studies have shown, the present negative results do not exclude the role of the *MTNR1B* polymorphisms studied in the development of myocardial infarction. Rather, they may indicate that *MTNR1B* polymorphisms are a minor risk factor for myocardial infarction.

## 1. Introduction

Melatonin, a neurohormone produced by the pineal gland [1] and controlled by the hypothalamic suprachiasmatic nucleus (SCN) [2], regulates the circadian rhythm [3]. This pleiotropic hormone has several different functions [4], but regulating circadian rhythm, including sleep and wakefulness, is considered the most important one [5]. Previous studies confirmed its fundamental antioxidant effects since it scavenges free radicals and stimulates antioxidant enzymes and molecules to prevent radicals [6,7], besides having anti-inflammatory and possibly epigenetic regulatory functions [8,9,10]. Melatonin’s circadian rhythm of secretion [11], through increased secretion during the night and decreased secretion during the day, coordinates physiological processes and behavioral activities [2,3]. Melatonin’s effects on human diseases are numerous [6], mostly manifested through a membrane receptor [5]. Melatonin receptors are G-coupled proteins [8], and we distinguish three melatonin receptor subtypes, of which MT1 (melatonin receptor 1A, MTNR1A) and MT2 (melatonin receptor 1B, MTNR1B) have been identified in mammals, whereas MT3 (melatonin receptor 1C, MTNR1C) has been identified in amphibians and birds [5]. Melatonin action involves the activation of a high-affinity membrane receptor, melatonin receptor 1B [3], encoded by the *MTNR1B* gene [12].

Cardiovascular diseases (CVD) represent the leading cause of death worldwide, and metabolic disorders, such as diabetes, are among the most important risk factors [13,14]. Genetic risk factors for diabetes and its complications, such as insulin resistance and vascular calcification, are associated with cardiovascular damage [15,16]. Diabetic complications are highly prevalent and may facilitate atherosclerosis and plaque progression, consequently leading to harmful coronary events [17,18]. Myocardial infarction (MI), one of CVD’s most important diagnostic subgroups, is the major cause of death in diabetic patients [12,17]. Numerous studies confirmed the association between *MTNR1B* gene polymorphisms and the risk of type 2 diabetes [2,6,15,19,20].

Melatonin production decreases with age, but this decrease in melatonin production is also observed in many age-related diseases, including cardiovascular diseases [8]. Melatonin’s important anti-aging function [5], besides the heart, affects many other organs through the activation of the MTNR1B receptor [19]. MTNR1B has a more important function in the cardiac phenotype compared with MTNR1A [5]. It should be emphasized that melatonin’s strong cardioprotective role [21] for stroke and myocardial infarction [3] depends on direct free radical scavenging and antioxidant activity, as well as on the stimulation of autophagic cell renewal, regulation of immune and inflammatory responses, improvement of mitochondrial function, and relief of endoplasmic reticulum stress [3,22,23]. It protects against myocardial ischemia, reducing myocardial fibrosis, improving myocardial calcium homeostasis, and alleviating cardiac arrhythmias [6].

Melatonin plays an important role in proper signal transduction through various receptors, including membrane and nuclear receptors, including the melatonin receptor, nuclear retinoid orphan receptors (ROR), tumor necrosis factor receptor (TNF), toll-like receptors (TLRs), and the Notch receptor [24]. An important melatonin signaling pathway in cardioprotection includes reperfusion injury salvage kinase (RISK), survivor activating factor enhancement (SAFE), and the Notch pathway. The association with downstream signaling molecules is important in the above pathways, in which sirtuin 1 (SIRT1) and SIRT3 contribute to melatonin protecting the heart from ischemic injury. The RISK signaling pathway is important in preventing cell apoptosis because melatonin reduces the transfer of Bax to mitochondria [22]. Another important signaling pathway of melatonin is the SAFE signaling pathway, in which melatonin phosphorylates JAK2-STAT3 kinase via the TNF receptor at the cell membrane, which stimulates expression of the *Bcl-2* gene and inhibits the *Bax* gene and other genes related to the induction of apoptosis. In this pathway, SIRT1, activated by melatonin, also regulates oxidative stress in cardiomyocytes and apoptosis by enhancing *Bcl-2* gene expression and inhibiting *Bax* and caspase-3 [22]. One of the important signaling pathways of melatonin in cardioprotection is its action via TLR, which reduces the size of myocardial infarction by activating the SAFE signaling pathway [23]. In addition to the RISK signaling pathway to inhibit apoptosis, melatonin can inhibit apoptosis and limit oxidative damage by signaling through the Notch receptor to protect cardiomyocytes during ischemia. Notch signaling modulates cell proliferation and apoptosis in various tissues [23]. Moreover, nuclear melatonin receptors belong to the orphan nuclear retinoic acid receptor family, and RORα has an endogenous protective function against myocardial infarction. Thus, research has shown that rats lacking RORα receptors are significantly more likely to suffer myocardial infarction and have higher levels of cardiac dysfunction [22]. The cardioprotective properties of melatonin are also related to the above-mentioned signaling pathways and its antioxidant effect on the duration of myocardial repolarization. In addition, melatonin has an antiarrhythmic effect related to its action on ventricular activation, independent of its antioxidant effect [24].

Apart from the circadian hormone melatonin, CVD is also affected by the circadian phenotype, named chronotype [25]. Chronotype represents a measure of preferred sleep time and activity related to wakefulness. We distinguish three categories of a person’s chronotype—evening type, morning type, and people who do not belong to any of the above categories or neither type [25,26]. Chronotype is related to the circadian rhythm of melatonin [27] and some CVD risk factors, such as hypertension and diabetes [11]. In addition, the evening chronotype is associated with higher morbidity, including higher rates of metabolic disorders and CVD [28] and higher mortality from CVD [25]. Research has also confirmed the association between chronotype and *MTNR1B* gene polymorphisms. The G allele of the rs10830963 polymorphism is associated with altered melatonin secretion and circadian phenotypes [2], and the evening type is associated with a higher risk of diabetes in individuals with the GG genotype of the rs10830963 *MITNR1B* gene polymorphism [27]. *MITNR1B* gene polymorphisms are associated with CVD risk factors such as type 2 diabetes. Moreover, cardiovascular diseases follow a circadian pattern of occurrence, and myocardial infarction most commonly occurs early in the morning or late in the evening. It has been shown that a person’s chronotype can influence the occurrence of myocardial infarction [29]. In addition, melatonin levels fluctuate daily, and there might be a link between chronotype and *MTNR1B* gene polymorphisms, as the G allele of *MTNR1B* rs10830963 is associated with increased *MTNR1B* mRNA expression [3]. Increased levels of *MTNR1B* mRNA result in higher melatonin signaling levels [3]. In addition, the G allele is associated with a longer duration of elevated melatonin levels, a delayed phase of the circadian melatonin shift, and a delayed light-dark cycle [30]. Therefore, the *MTNR1B* gene polymorphisms might affect the synchronization between the melatonin phase and the light-dark cycle, which is associated with chronotype.

In this case-control study, we studied the association between melatonin receptor 1B gene polymorphisms and susceptibility to myocardial infarction in surviving patients. For this purpose, differences between patients and control participants in the frequency of individual polymorphisms and genotypes were analyzed. In addition, genotype models of *MTNR1B* gene polymorphisms and possible haplotypes were analyzed. Furthermore, it was investigated whether CVD risk factors were associated with the analyzed *MTNR1B* gene polymorphisms. Specifically, the standardized and validated Horne-Östberg Morningness-Eveningness Questionnaire (MEQ) was used to test whether participants’ chronotype was associated with the *MTNR1B* gene polymorphisms, as well as the association of chronotype with CVD risk factors in MI patients. Previous studies showed that type 2 diabetes significantly affects myocardial infarction risk, and that *MTNR1B* gene polymorphisms are associated with increased diabetes risk, that the same receptor has a stronger protective effect against myocardial ischemia, and that *MTNR1B* gene polymorphisms are associated with circadian phenotype—chronotype. We aimed to investigate the association of *MTNR1B* polymorphisms with chronotype and susceptibility to myocardial infarction.

## 2. Results

### 2.1. Characteristics of the Study Participants

The baseline characteristics of the study participants are shown in Table 1. There were a total of 397 participants, 199 MI cases, and 198 controls. The distribution according to gender was similar between the groups. Cases were significantly older than controls. The mean diastolic blood pressure was significantly higher in the control group. However, most MI cases had hypertension and a significantly higher body mass index (BMI) than control participants. There were more smokers among the control participants. The prevalence of type 2 diabetes, previous CVD, and family history of CVD was significantly higher in MI patients than in control participants.

### 2.2. Genetic Analyses

The analyzed genotype frequencies of polymorphisms were consistent with Hardy-Weinberg equilibrium (*p* > 0.05). In addition, the minor allele frequencies of all analyzed SNPs were consistent with a HapMap phase 3 CEPH reference population. Table 2 shows the distribution of genotypes and alleles of *MTNR1B* polymorphisms.

There were no significant associations between rs10830963, rs1387153, and rs4753426 in the *MTNR1B* gene and MI. Genotype model analyses (recessive, dominant, and codominant) were also not significantly different between cases and controls (Supplementary Table S1).

Table 3 shows the results of the logistic regression model fitted to estimate the independent effect of the analyzed polymorphism after adjustment for cardiovascular risk factors. SNP rs1387153 was associated with an increased risk of MI in the present study. In the recessive model, CC versus CT + TT, the *p*-value was 0.043 (OR = 0.30; 95% CI 0.09–0.96). A logistic regression model showed a significant association with age (*p* = 0.028), diastolic blood pressure (*p* = 0.001), systolic blood pressure (*p* = 0.003), BMI (*p* = 0.003), smoking (*p* < 0.001), and previous CVD (*p* < 0.001).

The frequency of predicted haplotypes in MI patients and control participants is shown in Table 4. A statistically significant difference in haplotype distribution was confirmed for the CTT haplotype (*p* = 0.015) between MI patients and control participants. However, after correction for multiple tests, this difference was no longer significant (q = 0.060). There is linkage disequilibrium (LD) for all analyzed polymorphisms in the *MTNR1B* gene. The linkage disequilibrium between rs10830963 and rs1387153 was D’ = 0.842 and r^2^ = 0.701; between rs10830963 and rs4753426 was D’ = 0.945 and r^2^ = 0.368; and LD between rs1387153 and rs4753426 was D’ = 0.819 and r^2^ = 0.273.

An association was found between some cardiovascular risk factors and *MTNR1B* gene polymorphisms in patients with MI (Table 5). The rs10830963 and rs1387153 polymorphisms were associated with type 2 diabetes mellitus and previous CVDs.

### 2.3. Analysis of Chronotype and Polymorphisms

Analysis between MI patients and control participants revealed no association between chronotype and any *MTNR1B* polymorphisms tested at the allele level (Supplemental Table S2). The association between *MTNR1B* genotype frequency and chronotype in MI patients is shown in Table 6. There was no significant difference between individual genotypes and specific chronotypes in MI patients.

Table 7 shows the association between cardiovascular risk factors and overall chronotype score and chronotype in MI patients. Only one patient belongs to the evening chronotype, so it was not included in the analysis.

## 3. Discussion

Our results of the analysis of *MTNR1B* gene polymorphisms (rs10830963, rs1387153, and rs4753426) did not confirm the association between the analyzed variations of the melatonin receptor 1B gene and MI susceptibility. In addition, an association study was performed to determine which *MTNR1B* gene variants might be associated with chronotype in MI patients, and no association between *MTNR1B* gene variants and chronotype was found.

To the best of our knowledge, there are no comparable studies, so it is difficult to reconcile the results of this study with studies in patients with diabetes. However, the study by Huber et al. in hypertensive patients showed that the effects of *MTNR1B* gene polymorphisms and haplotypes did not differ between patients with and without myocardial infarction [6]. Moreover, although the frequency of the CTT haplotype was significantly different between the MI patient group and the control group in the present study, this difference was no longer significant after correction for multiple testing. Similarly, a study by Xue et al. showed that *MTNR1B* gene variations in patients with diabetes did not increase their risk of CVD mortality [12]. In addition, some of the analyzed cardiovascular risk factors, such as diastolic and systolic blood pressure, BMI, and smoking, were significantly associated with the analyzed polymorphisms in MI patients in this study. However, no significant association was found for the rs4753426 polymorphism in the present study. In addition, the rs10830963 and rs1387153 polymorphisms were associated with type 2 diabetes in MI patients, which is consistent with previous studies [31,32,33,34]. Furthermore, no association was found between chronotype and *MTNR1B* gene variants in individuals with myocardial infarction in the present study, and other studies also showed that chronotype was not significantly associated with increased risk of cardiovascular diseases or myocardial infarction [28,30,35,36].

Our results indicate no association between *MTNR1B* gene variants and myocardial infarction, unlike other studies that confirmed an association with increased LDL and triglyceride levels and arterial stiffness in people without CVD [37]. In addition, variations in the *MTNR1B* gene have been associated with its expression and signaling changes [6]. A commonly studied polymorphism of the *MTNR1B* gene is rs108390963, which is associated with an increased risk of diabetes. Diabetic patients who are carriers of the minor G allele have a 19% higher risk of myocardial infarction [12,17]. However, the minor G allele has not been associated with increased mortality from myocardial infarction or other cardiovascular diseases in people with diabetes [12]. In addition, the minor G allele has been shown to be associated with increased melatonin levels and disrupted circadian rhythms, i.e., a delayed light-dark cycle [3]. Indeed, the minor G allele rs10830963 is associated with increased mRNA expression of the *MTNR1B* gene and, thus, with increased melatonin levels [3]. Since melatonin controls circadian rhythm, increased melatonin secretion can lead to circadian rhythm disturbances associated with cardiovascular and metabolic disorders [3,38,39,40].

A decrease in melatonin levels is associated with aging and is especially pronounced in some age-related diseases [41]. It is important to emphasize that melatonin levels have been reduced in cardiovascular diseases such as coronary artery disease, arterial hypertension, heart failure, and after myocardial infarction [37]. Previous studies confirmed decreased melatonin production in patients with coronary artery disease [42]. In contrast, melatonin synthesis increases in response to myocardial infarction, supporting the role of melatonin in protecting the heart after infarction [42]. Activation of the MTNR1B receptor on myocytes by melatonin leads to the attenuation of ischemic injury to the heart [19]. Animal studies have shown that melatonin inhibits the formation of reactive oxygen species and promotes the intracellular accumulation of calcium ions, which protects myocytes from damage caused by ischemia [8]. When melatonin was administered to patients with myocardial infarction shortly after the onset of initial symptoms, there was a significant reduction in infarct size [5]. Moreover, melatonin is a ligand for nuclear retinoid orphan receptors, including the RORα receptor, one of the circadian clock proteins involved in the control of the circadian rhythm itself [4]. In addition to the already known cardioprotective functions of melatonin, its association with the RORα receptor may be another way to protect against ischemic damage to the myocardium [4,43].

The association between *MTNR1B* gene variations and chronotype has also been studied, mainly in diabetic patients. The aging process affects chronotype, and older people tend to be morning types [35], and we also obtained similar results. The only association found in this study for a specific chronotype category was that neither type had significantly higher systolic pressure. However, no association was found between chronotype and *MTNR1B* gene variants in individuals with myocardial infarction. Thus, individuals belonging to the morning type and carriers of the minor G allele rs10830963 have been shown to have a higher risk of developing diabetes [27,36]. However, it is thought that a shift in circadian rhythm due to short sleep duration and early morning food intake increases the risk of diabetes, not *MTNR1B* gene variations [36]. On the other hand, another study showed that the minor G allele was associated with delayed melatonin secretion and an evening chronotype [3]. Shift work significantly affects an individual’s chronotype, and some studies have shown that the G allele of the rs10830963 polymorphism is associated with a reduced risk of stroke. In contrast, the CC genotype of the same polymorphism has been associated with a higher risk of stroke in night shift workers [3]. However, another study showed that carriers of risk allele G of the rs10830963 polymorphism who work shift work or have a morning or evening chronotype do not have an increased risk of type 2 diabetes. In the aforementioned study, the authors showed that night shift work and morning or evening preference were associated with the prevalence of type 2 diabetes, but the rs10830963 polymorphism was not [36].

### Study Limitations

This study has several limitations, but sample size is one of the most important. In previous studies of *MTNR1B* gene polymorphisms, the measure of association as estimated by OR ranged from 0.97 to 1.25 [44] and can be considered a small effect. Although the magnitude of the effect size is very likely, a larger number of participants could lead to a significant association between genotype and phenotype. In addition, the analyzed polymorphisms of the *MTNR1B* gene, which were selected based on previous publications, can be considered a limitation of the study. The selected polymorphisms were mainly associated with type 2 diabetes and fasting blood glucose levels, and the lack of association can be partly explained by the low prevalence of MI patients with diabetes—22.1%. In addition, because of the low frequency of some genotypes, it was not possible to statistically estimate associations. Similarly, we cannot exclude the possibility that statistically significant differences in baseline characteristics, such as hypertension and dyslipidemia, may influence the outcome of the study. The reproducibility of association studies is low, mainly because of the small effect size of individual polymorphisms. This explains the difference between our results and those of most other studies on the *MTNR1B* gene polymorphism. Circadian phenotype assessment and chronotype classification were based on a self-reported questionnaire and could be prone to bias. Nevertheless, this could not significantly affect the outcome of the study. In addition, because of the limited data collected retrospectively, we could not assess the potential influence of shift work schedules on chronotype.

The way cases were defined could introduce bias in the research. Since ischemic ECG changes were on the list of criteria that needed to be fulfilled, such a definition of outcome could potentially exclude non-STEMI patients. Myocardial infarction, as well as some other cardiovascular diseases, represent a continuum rather than a bivariant category. Still, the implementation of guidelines into clinical practice requires a definition of categories and the loss of some information in the process. Although no cardiovascular diseases were detected in the control group at the time of the study, we cannot exclude the possibility of cardiovascular events occurring later in life. However, the advantage of this study is the homogeneity of the sample in terms of demographic variables such as gender, ethnicity, and social environment. University Hospital Osijek is a tertiary-level medical center with a large catchment area of 258,026 inhabitants [45], and all patients with MI are referred to this center. Since this is a publicly funded hospital and there are no private hospitals in this county, the possible influence of social stratification can be excluded.

## 4. Materials and Methods

### 4.1. Participants

This case-control study was based on data collected in previously published studies of circadian clock variations in myocardial infarction patients [46,47,48]. The sample size consisted of 199 cases, 114 men and 85 women, with nonfatal acute myocardial infarction hospitalized at the Clinical Department of Cardiovascular Diseases and Intensive Care, University Hospital Osijek, Croatia, from 1 August 2012 to 31 December 2018. We reviewed the medical records of all participants.

Cases were included if they fulfilled the criteria for type 1 or type 2 MI, according to Thygesen et al. [49,50]. At least two out of three criteria had to be met: cardiac troponin T above the 99th percentile, chest pain symptoms for more than 30 min indicating ischemia, and ischemic electrocardiographic (ECG) changes.

The control group consisted of 198 participants, 103 men and 95 women, with no cardiovascular diseases noted in medical records. They were selected after a check-up by their primary care physician in the outpatient clinic.

Participants were excluded if they had severe heart disease or type 2 diabetes mellitus or were related to other participants in the study since chronotype traits are heritable [51,52]. Blood pressure in mmHg, height, and weight were measured in all participants.

The Ethics Committee of the University Hospital Osijek (No. 25-1:3160-3/2012) approved this study. It was performed in accordance with the Declaration of Helsinki and its revisions.

### 4.2. Questionnaires

Participants were interviewed about their medical history with a questionnaire that had information about age, sex, smoking status (current smoker or no-smoker), family history of CVD, previous CVDs, hypertension, dyslipidemia, and type 2 diabetes mellitus. A family history of CVD was defined as angina pectoris, coronary artery bypass graft, and MI in first-degree relatives before age 60 years. Previous cardiovascular diseases were defined as angina pectoris, MI, arrhythmia, or stroke. Individual chronotype was determined using the previously published and validated Croatian version of the Horne-Östberg Morningness-Eveningness Questionnaire (MEQ) [53,54,55]. The MEQ contains 19 questions, and according to the total score, participants were categorized into the following types: morning (59–86), neither (42–58), and evening (16–41). Participants with missing chronotype information were excluded.

### 4.3. Polymorphysm Selection and Genotyping

Genotyping was performed using pre-designed TaqMan^®^ assays on the Applied Biosystems QuantStudio 5 real-time PCR system. Three single nucleotide polymorphisms (SNPs: rs10830963, rs1387153, and rs4753426) in the *MTNR1B* gene were analyzed using QuantStudio Design & Analysis Software version 1.5.2 (Applied Biosystems, Foster City, CA, USA). The above polymorphisms were selected because they had previously been associated with myocardial infarction risk, chronotype, arterial stiffness, and cardiovascular risk factors, particularly type 2 diabetes mellitus [2,19,27,33,37]. The polymorphisms analyzed are all located in the intronic region of the *MTNR1B* gene. Participants with missing genotyping data were excluded.

The SNPStats web tool was used to analyze recessive, dominant, and codominant genotype models independently [56]. In addition, the web tool SHEsis was used to reconstruct haplotypes from genotype data [57,58].

### 4.4. Statistical Analyses

Descriptive statistics were used to present data as numbers and percentages or as arithmetic means ± standard deviations. The chi-square test was used to calculate a significant difference for each polymorphism to compare allele frequency and genotype distribution between MI patients and control participants. Genotyping quality control was also performed using the chi-square test for goodness-of-fit and an analysis of genotype distributions compared with those expected by Hardy-Weinberg equilibrium. Logistic regression was employed to estimate the effect of *MTNR1B* rs10830963, rs1387153, and rs4753426 genotypes on the probability of myocardial infarction in patients. In addition, age, diastolic and systolic blood pressure, BMI, smoking, hypertension, dyslipidemia, family history of CVD, and previous CVDs were used as covariates. The chi-square test was used to determine the relationship between cardiovascular risk variables and *MTNR1B* polymorphisms. In addition, the chi-square test and Kruskal-Wallis test were used to determine the significance of the relationships between allele and genotype frequencies and chronotype. The association was considered significant if the *p*-value was equal to or less than 0.05. However, the Benjamini-Hochberg method (false discovery rate—FDR value) was used due to the three polymorphisms studied. Therefore, only q-values less than 0.05 were considered statistically significant. Statistical analyses were performed using SPSS software (version 26.0, SPSS Inc., Chicago, IL, USA).

## 5. Conclusions

Physiological processes are coordinated with the diurnal rhythm of the circadian hormone melatonin, for whose function the high-affinity membrane receptor MTNR1B is important. Through the MTNR1B receptor, melatonin plays an important role not only in the maintenance of cardiovascular homeostasis but also in the pathogenesis of disease. In this study, no association was found between *MTNR1B* gene variations (rs10830963, rs1387153, and rs4753426) and myocardial infarction because the effect size of a single gene on complex clinical parameters such as myocardial infarction is relatively small. In addition, no significant association was found between *MTNR1B* gene polymorphism and chronotype in patients with MI. However, some cardiovascular risk factors were associated with the analyzed polymorphisms and chronotypes in MI patients. Nevertheless, as some previous studies have shown, these negative results do not exclude the role of previously mentioned polymorphisms in the development of myocardial infarction. Rather, they may indicate that the *MTNR1B* gene polymorphisms are minor risk factors for susceptibility to myocardial infarction.

## Figures and Tables

**Table 1:** The baseline characteristics of study participants.

Risk Factors	MI (*n* = 199)	Ctrl (*n* = 198)	*p*-Value *
Age (years)	66.32 ± 12.35	61.98 ± 12.84	**0.002**
Gender (male, %)	114 (57.3%)	103 (52%)	0.293
Diastolic blood pressure (mmHg)	76.62 ± 9.97	79.65 ± 7.35	**0.004**
Systolic blood pressure (mmHg)	126.75 ± 15.87	125.94 ± 15.04	0.433
BMI (kg/m^2^)	28.74 ± 4.75	26.86 ± 4.21	**<0.001**
Gender (male)	114 (57.3%)	103 (52%)	0.293
Smoking (yes)	98 (49.2%)	151 (76.3%)	**<0.001**
Hypertension	106 (53.3%)	59 (29.8%)	**<0.001**
Dyslipidemia	26 (13.1%)	23 (11.6%)	0.661
Diabetes mellitus type 2	44 (22.1%)	0	**<0.001**
Family history of CVD	47 (23.6%)	27 (13.7%)	**0.011**
Previous CVD	142 (71.3%)	14 (7.1%)	**<0.001**

* Mann-Whitney U test. MI—myocardial infarction patients; Ctrl—controls; BMI—body mass index; CVD—cardiovascular diseases. A family history of CVD was defined as evidence of coronary artery disease in first-degree relatives before 60 years of age. Previous CVD was defined as a reported history of angina pectoris or arrhythmia. Significant values are bolded.

**Table 2:** Allele and genotype distribution and frequencies of the *MTNR1B* polymorphisms.

SNP	Minor Allele	MAF * MI	MAF * Ctrl	OR (95% CI)	*p*-Value ^†^	Genotype	Genotype Frequency (%)			
							MI	Ctrl	*p*-Value ^†^	Χ^2^
rs10830963	G	0.294	0.298	1.02 (0.75–1.38)	0.902	CC	48.7	50.0	0.676	0.78
						CG	43.7	40.4		
						GG	7.5	9.6		
rs1387153	T	0.304	0.283	0.90 (0.67–1.23)	0.512	CC	48.7	52.0	0.801	0.44
						CT	41.7	39.4		
						TT	9.5	8.6		
rs4753426	T	0.490	0.500	1.04 (0.79–1.38)	0.777	CC	26.6	24.7	0.905	0.20
						CT	48.7	50.5		
						TT	24.6	24.7		

* MAF—minor allele frequency; † Chi-square test. MI—myocardial infarction patients; Ctrl—controls.

**Table 3:** A logistic regression model for myocardial infarction adjusted for cardiovascular risk factors.

Risk Factors	OR (95% CI)	*p*-Value
Age	1.02 (0.99–1.05)	**0.028**
Diastolic blood pressure	0.89 (0.83–0.96)	**0.001**
Systolic blood pressure	1.06 (1.02–1.11)	**0.001**
BMI	1.15 (1.0–1.26)	**0.004**
Smoking (yes)	4.33 (2.27–8.27)	**<0.001**
Hypertension	0.89 (0.39–2.03)	0.784
Dyslipidemia	0.67 (0.23–1.95)	0.464
Family history of CVD	1.37 (0.77–2.43)	0.287
Previous CVD	0.07 (0.03–0.16)	**<0.001**
rs1387153 (CC)	0.30 (0.09–0.96)	**0.043**

BMI—body mass index; OR—odds ratio. Statistically significant values are bolded.

**Table 4:** Frequencies and distribution of probable MTNR1B haplotypes in the patients and the control groups.

rs10830963	rs1387153	rs4753426	Frequency MI	Frequency Ctrl	OR (95% CI)	*p*-Value	q-Value
C	C	C	0.21	0.20	1.01 (0.71–1.42)	0.976	0.976
C	C	T	0.45	0.48	0.87 (0.66–1.16)	0.344	0.459
C	T	C	0.02	0.01	2.38 (0.56–10.09)	0.227	0.454
C	T	T	0.03	0.01	3.88 (1.19–12.56)	0.015	0.060

MI—myocardial infarction patients; Ctrl—controls; q-value corrections of significance values were applied using the Benjamini-Hochberg correction method (false-discovery rate—FDR values).

**Table 5:** The association between cardiovascular risk factors and *MTNR1B* polymorphisms in MI patients.

Risk Factors	rs10830963	rs1387153	rs4753426
Age (years)	0.144	0.082	0.430
Diastolic blood pressure (mmHg)	0.725	0.454	0.068
Systolic blood pressure (mmHg)	0.408	0.070	0.152
BMI (kg/m^2^)	0.377	0.110	0.622
Gender (male)	0.605	0.147	0.530
Smoking (yes)	0.992	0.733	0.994
Hypertension	0.056	0.363	0.432
Dyslipidemia	0.221	0.901	0.051
Diabetes mellitus type 2	**0.044**	**0.002**	0.630
Family history of CVD	0.469	0.395	0.928
Previous CVD	**0.011**	**0.033**	0.379

Chi-square test *p*-value. BMI—body mass index; CVD—cardiovascular diseases. The bold values are statistically significant.

**Table 6:** The association between chronotype scores at the genotype level of *MNTR1B* polymorphisms.

SNPs	Genotype	Evening Type	Neither Type	Morning Type	*p*-Value *
rs10830963	CC	1 (100%)	46 (46%)	50 (51%)	0.136
	CG	0	49 (49%)	38 (38.8%)	
	GG	0	5 (5%)	10 (10.2%)	
rs1387153	CC	1 (100%)	46 (46%)	50 (51%)	0.544
	CT	0	45 (45%)	38 (38.8%)	
	TT	0	9 (9%)	10 (10.2%)	
rs4753426	CC	0	26 (26%)	27 (27.6%)	0.261
	CT	1 (100%)	49 (49%)	47 (48%)	
	TT	0	25 (25%)	24 (24.5%)	

* Kruskal-Wallis test.

**Table 7:** The association between cardiovascular risk factors and chronotype in MI patients.

Risk Factors	MEQ	Neither Type (n = 100)	Morning Type (n = 98)
Age (years)	**<0.001**	0.408	0.086
Diastolic blood pressure (mmHg)	0.991	0.955	0.992
Systolic blood pressure (mmHg)	0.606	**0.007**	0.967
BMI (kg/m^2^)	**0.030**	0.679	0.168
Gender (male)	0.187	0.103	0.720
Smoking	0.690	0.801	0.563
Hypertension	0.229	0.250	0.314
Dyslipidemia	0.486	0.664	0.616
Diabetes mellitus type 2	0.594	0.764	0.693
Family history of CVD	0.857	0.682	0.467
Previous CVD	0.089	0.408	0.098

Chi-square test *p*-value. BMI—body mass index; CVD—cardiovascular diseases. Bold are significant values.

## Data Availability

The dataset generated during the study is available from the corresponding author on reasonable request.

## Supplementary Materials

The supporting information can be downloaded at: https://www.mdpi.com/article/10.3390/ijms241411444/s1.

## References

[1] Azedi F., Mehrpour M., Talebi S., Zendedel A., Kazemnejad S., Mousavizadeh K., Beyer C., Zarnani A.H., Joghataei M.T. (2019). Melatonin regulates neuroinflammation ischemic stroke damage through interactions with microglia in reperfusion phase. Brain Res..

[2] Zhu H., Zhao Z., Liu H., Cai J., Lu Q., Ji L., Xu J. (2023). The melatonin receptor 1B gene links circadian rhythms and type 2 diabetes mellitus: An evolutionary story. Ann. Med..

[3] Chen Y., Yang L., Liang Y.Y., He Z., Ai Q.-Y.H.Y.H., Chen W., Xue H., Zhou M., Wang Y., Ma H. (2022). Interaction of night shift work with polymorphism in melatonin receptor 1B gene on incident stroke. Scand. J. Work. Environ. Health.

[4] Lochner A., Marais E., Huisamen B. (2018). Melatonin and cardioprotection against ischaemia/reperfusion injury: What’s new? A review. J. Pineal Res..

[5] Han D., Wang Y.Y., Chen J., Zhang J., Yu P., Zhang R., Li S., Tao B., Wang Y.Y., Qiu Y. (2019). Activation of melatonin receptor 2 but not melatonin receptor 1 mediates melatonin-conferred cardioprotection against myocardial ischemia/reperfusion injury. J. Pineal Res..

[6] Huber M., Treszl A., Reibis R., Teichmann C., Zergibel I., Bolbrinker J., Scholze J., Wegscheider K., Völler H., Kreutz R. (2013). Genetics of melatonin receptor type 2 is associated with left ventricular function in hypertensive patients treated according to guidelines. Eur. J. Intern. Med..

[7] Škrlec I. (2022). The Influence of Dental Implants on the Circadian Clock and the Role of Melatonin in the Oral Cavity. Explor. Res. Hypothesis Med..

[8] Dominguez-Rodriguez A., Abreu-Gonzalez P., Sanchez-Sanchez J.J., Kaski J.C., Reiter R.J. (2010). Melatonin and circadian biology in human cardiovascular disease. J. Pineal Res..

[9] Kim J.Y., Jelinek J., Lee Y.H., Kim D.H., Kang K., Ryu S.H., Moon H.R., Cho K., Rha S.H., Cha J.K. (2023). Hypomethylation in MTNR1B: A novel epigenetic marker for atherosclerosis profiling using stenosis radiophenotype and blood inflammatory cells. Clin. Epigenetics.

[10] Škrlec I. (2023). Circadian system microRNAs—Role in the development of cardiovascular diseases. Adv. Protein Chem. Struct. Biol..

[11] Estarlich M., Tolsa C., Trapero I., Buigues C. (2022). Circadian Variations and Associated Factors in Patients with Ischaemic Heart Disease. Int. J. Environ. Res. Public Health.

[12] Xue P., Tan X., Wu J., Tang X., Benedict C. (2022). No association between a common type 2 diabetes risk gene variant in the melatonin receptor gene (MTNR1B) and mortality among type 2 diabetes patients. J. Pineal Res..

[13] Hukic D.S., Lavebratt C., Olsson E., Östenson C.G., Eriksson S.V., Erlinge D., Schalling M., Ösby U. (2017). Troponin T levels associated with genetic variants in NOTCH2 and MTNR1B in women with psychosis. Psychiatry Res..

[14] Škrlec I., Talapko J., Džijan S., Cesar V., Lazić N., Lepeduš H. (2022). The Association between Circadian Clock Gene Polymorphisms and Metabolic Syndrome: A Systematic Review and Meta-Analysis. Biology.

[15] Sousa A.G., Selvatici L., Krieger J.E., Pereira A.C. (2011). Association between genetics of diabetes, coronary artery disease, and macrovascular complications: Exploring a common ground hypothesis. Rev. Diabet. Stud..

[16] Goodarzi M.O., Rotter J.I. (2020). Genetics Insights in the Relationship between Type 2 Diabetes and Coronary Heart Disease. Circ. Res..

[17] Cui J., Liu Y., Li Y., Xu F., Liu Y. (2021). Type 2 Diabetes and Myocardial Infarction: Recent Clinical Evidence and Perspective. Front. Cardiovasc. Med..

[18] Yuan T., Yang T., Chen H., Fu D., Hu Y., Wang J., Yuan Q., Yu H., Xu W., Xie X. (2019). New insights into oxidative stress and inflammation during diabetes mellitus-accelerated atherosclerosis. Redox Biol..

[19] Tan X., Benedict C. (2020). Increased Risk of Myocardial Infarction Among Patients with Type 2 Diabetes Who Carry the Common rs10830963 Variant in the MTNR1B Gene. Diabetes Care.

[20] Rasmussen-Torvik L.J., Guo X., Bowden D.W., Bertoni A.G., Sale M.M., Yao J., Bluemke D.A., Goodarzi M.O., Chen Y.I., Vaidya D. (2012). Fasting Glucose GWAS Candidate Region Analysis across Ethnic Groups in the Multi-Ethnic Study of Atherosclerosis (MESA). Genet. Epidemiol..

[21] Genade S., Genis A., Ytrehus K., Huisamen B., Lochner A. (2008). Melatonin receptor-mediated protection against myocardial ischaemia/reperfusion injury: Role of its anti-adrenergic actions. J. Pineal Res..

[22] Fu Z., Jiao Y., Wang J., Zhang Y., Shen M., Reiter R.J., Xi Q., Chen Y. (2020). Cardioprotective Role of Melatonin in Acute Myocardial Infarction. Front. Physiol..

[23] Randhawa P.K., Gupta M.K. (2020). Melatonin as a protective agent in cardiac ischemia-reperfusion injury: Vision/Illusion?. Eur. J. Pharmacol..

[24] Sedova K.A., Bernikova O.G., Cuprova J.I., Ivanova A.D., Kutaeva G.A., Pliss M.G., Lopatina E.V., Vaykshnorayte M.A., Diez E.R., Azarov J.E. (2019). Association Between Antiarrhythmic, Electrophysiological, and Antioxidative Effects of Melatonin in Ischemia/Reperfusion. Int. J. Mol. Sci..

[25] Chellappa S.L., Vujovic N., Williams J.S., Scheer F.A.J.L. (2019). Impact of Circadian Disruption on Cardiovascular Function and Disease. Trends Endocrinol. Metab..

[26] Roenneberg T., Kuehnle T., Juda M., Kantermann T., Allebrandt K., Gordijn M., Merrow M. (2007). Epidemiology of the human circadian clock. Sleep Med. Rev..

[27] Tan X., Ciuculete D.M., Schiöth H.B., Benedict C. (2020). Associations between chronotype, MTNR1B genotype and risk of type 2 diabetes in UK Biobank. J. Intern. Med..

[28] Knutson K.L., von Schantz M. (2018). Associations between chronotype, morbidity and mortality in the UK Biobank cohort. Chronobiol. Int..

[29] Selvi Y., Smolensky M.H., Boysan M., Aydin A., Besiroglu L., Atli A., Gumrukcuoglu H.A. (2011). Role of patient chronotype on circadian pattern of myocardial infarction: A pilot study. Chronobiol. Int..

[30] Lane J.M., Chang A.M., Bjonnes A.C., Aeschbach D., Anderson C., Cade B.E., Cain S.W., Czeisler C.A., Gharib S.A., Gooley J.J. (2016). Impact of Common Diabetes Risk Variant in MTNR1B on Sleep, Circadian, and Melatonin Physiology. Diabetes.

[31] Heshmat T.S., Kareem H.S., Khalil N.K.M., Shaker O.G. (2014). The association between the melatonin receptor 1B gene polymorphism rs10830963 and glucose levels in type 2 diabetes. Egypt. J. Intern. Med..

[32] Patel R., Rathwa N., Palit S.P., Ramachandran A.V., Begum R. (2018). Association of melatonin & MTNR1B variants with type 2 diabetes in Gujarat population. Biomed. Pharmacother..

[33] Shen L.L., Jin Y. (2019). Effects of MTNR1B genetic variants on the risk of type 2 diabetes mellitus: A meta-analysis. Mol. Genet. Genom. Med..

[34] Bouatia-Naji N., Bonnefond A., Cavalcanti-Proença C., Sparsø T., Holmkvist J., Marchand M., Delplanque J., Lobbens S., Rocheleau G., Durand E. (2009). A variant near MTNR1B is associated with increased fasting plasma glucose levels and type 2 diabetes risk. Nat. Genet..

[35] Škrlec I., Milić J., Heffer M., Steiner R., Peterlin B., Wagner J. (2018). Association of Circadian Rhythm with Myocardial Infarction. Acta Clin. Croat..

[36] Dashti H.S., Vetter C., Lane J.M., Smith M.C., Wood A.R., Weedon M.N., Rutter M.K., Garaulet M., Scheer F.A.J.L., Saxena R. (2020). Assessment of MTNR1B Type 2 diabetes genetic risk modification by shift work and morningness-eveningness preference in the UK biobank. Diabetes.

[37] Kolomeichuk S.N., Korneva V.A., Kuznetsova T.Y., Korostovtseva L.S., Bochkarev M.V., Sviryaev Y.V., Blagonravov M.L. (2023). MTNR1A and MTNR1B Gene Variants of the Melatonin Receptor and Arterial Stiffness in Persons without Arterial Hypertension. Bull. Exp. Biol. Med..

[38] Škrlec I., Milić J., Cilenšek I., Petrovič D., Wagner J., Peterlin B. (2019). Circadian clock genes and myocardial infarction in patients with type 2 diabetes mellitus. Gene.

[39] Ma H., Liu G., Guo L., Geng Q. (2017). The link between brain and heart in mental stress-induced myocardial ischemia. Heart Mind.

[40] Škrlec I., Milić J., Steiner R. (2020). The Impact of the Circadian Genes CLOCK and ARNTL on Myocardial Infarction. J. Clin. Med..

[41] Cardinali D.P., Hardeland R. (2017). Inflammaging, Metabolic Syndrome and Melatonin: A Call for Treatment Studies. Neuroendocrinology.

[42] Dominguez-Rodriguez A., Abreu-Gonzalez P., Avanzas P. (2012). The role of melatonin in acute myocardial infarction. Front. Biosci..

[43] De Seabra M.L.V., Bignotto M., Pinto L.R., Tufik S. (2000). Randomized, double-blind clinical trial, controlled with placebo, of the toxicology of chronic melatonin treatment. J. Pineal Res..

[44] Li Y.Y., Wang H., Zhang Y.Y. (2022). Melatonin receptor 1B gene rs10830963 C/G polymorphism associated with type 2 diabetes mellitus: An updated meta-analysis of 13,752 participants. Heliyon.

[45] Imširović A., Kraljik N. (2022). Natural Change in Population in Osijek-Baranja County in 2021.

[46] Škrlec I., Milic J., Heffer M., Peterlin B., Wagner J. (2018). Genetic variations in circadian rhythm genes and susceptibility for myocardial infarction. Genet. Mol. Biol..

[47] Škrlec I., Talapko J., Juzbašić M., Steiner R. (2021). Sex Differences in Circadian Clock Genes and Myocardial Infarction Susceptibility. J. Cardiovasc. Dev. Dis..

[48] Škrlec I., Milić J., Heffer M., Wagner J., Peterlin B. (2019). Circadian clock genes and circadian phenotypes in patients with myocardial infarction. Adv. Med. Sci..

[49] Thygesen K., Alpert J.S., Jaffe A.S., Simoons M.L., Chaitman B.R., White H.D., Katus H.A., Lindahl B., Morrow D.A., Clemmensen P.M. (2012). Third universal definition of myocardial infarction. Circulation.

[50] Thygesen K., Alpert J.S., Jaffe A.S., Chaitman B.R., Bax J.J., Morrow D.A., White H.D., Mickley H., Crea F., Van De Werf F. (2019). Fourth universal definition of myocardial infarction (2018). Eur. Heart J..

[51] Portaluppi F., Tiseo R., Smolensky M.H., Hermida R.C., Ayala D.E., Fabbian F. (2012). Circadian rhythms and cardiovascular health. Sleep Med. Rev..

[52] Carpen J.D., Archer S.N., Skene D.J., Smits M., von Schantz M. (2005). A single-nucleotide polymorphism in the 5′-untranslated region of the hPER2 gene is associated with diurnal preference. J. Sleep Res..

[53] Horne J.A., Ostberg O. (1976). A self-assessment questionnaire to determine morningness-eveningness in human circadian rhythms. Int. J. Chronobiol..

[54] Horne J., Ostberg O. (1977). Individual differences in human circadian rhythms. Biol. Psychol..

[55] Milić J., Škrlec I., Milić Vranješ I., Matić M., Sertić D., Heffer M. (2018). The Croatian Translation of the Horne and Östberg Morningness-Eveningness Questionnaire With a Brief Review of Circadian Typology. Southeast. Eur. Med. J..

[56] Solé X., Guinó E., Valls J., Iniesta R., Moreno V. (2006). SNPStats: A web tool for the analysis of association studies. Bioinformatics.

[57] Shi Y.Y., He L. (2005). SHEsis, a powerful software platform for analyses of linkage disequilibrium, haplotype construction, and genetic association at polymorphism loci. Cell Res..

[58] Li Z., Zhang Z., He Z., Tang W., Li T., Zeng Z., He L., Shi Y. (2009). A partition-ligation-combination-subdivision EM algorithm for haplotype inference with multiallelic markers: Update of the SHEsis (http://analysis.bio-x.cn). Cell Res..

