# Supplementary material for: Myocardial Infarction Susceptibility and the MTNR1B Polymorphisms
Source: Int J Mol Sci. 2023 Jul 14;24(14):11444. doi: 10.3390/ijms241411444 (PMC10380655; doi:10.3390/ijms241411444)
Supplement: Supplementary file 1 [file ijms-24-11444-s001.zip › ijms-2493215-supplementary.pdf]

Supplementary data

Myocardial infarction susceptibility and the *MTNR1B* polymorphisms

Table S1. Genotype models of the *MTNR1B* gene polymorphisms.

**SNPs**	**Dominant model**			**Recessive model**			**Codominant model**		
	p	OR	95% CI	p	OR	95% CI	p	OR	95% CI
rs10830963	0.802	0.95	0.64-1.41	0.465	1.30	0.64-2.64	0.680	0.90	0.60-1.36
rs1387153	0.514	0.88	0.56-1.30	0.626	0.84	0.42-1.67	0.801	0.89	0.58-1.34
rs4753426	0.667	1.10	0.70-1.73	0.977	1.01	0.94-1.59	0.902	1.12	0.69-1.80

P values shown in the table are corrected for the multiple comparisons.

Table S2. The association between self-reported chronotype and analyzed *MNTR1B* polymorphisms at allele level.

**Polymorphisms**	**MI patients (*n*=199)**	**Controls (*n*=198)**
rs10830963	0.091	0.963
rs1387153	0.612	0.852
rs4753426	0.885	0.297

Chi-square test p-value.
